# Hybrid operation for arteriovenous malformations with associated multiple intracranial aneurysms and subarachnoid hemorrhage: Case report

**DOI:** 10.1097/MD.0000000000028944

**Published:** 2022-02-25

**Authors:** Fei Xie, Lin Huang, Yongqiang Ye, Jianqiang Hao, Janwei Lv, Seidu A. Richard

**Affiliations:** a Department of Neurosurgery, The First People's Hospital of Ziyang, No. 66, Rende west road, Ziyang, Sichuan, PR China; b Department of Cardiology, The First People's Hospital of Ziyang, No. 66, Rende west road, Ziyang, Sichuan, PR China; c Department of Medicine, Princefield University, Ho-Volta Region, Ghana, West Africa.

**Keywords:** aneurysm, arteriovenous malformations, brain, hybrid, intracranial, subarachnoid hemorrhage

## Abstract

**Rationale:**

The hybrid surgical concept for the treatment of brain arteriovenous malformations (AVMs) with associated intracranial aneurysms (IAs) is still not widely practiced. Concomitant occurrence of AVMs with IAs is common. Subarachnoid hemorrhage (SAH) as a result of AVM or IA rupture is often associated with these dual pathological phenomena. We present a case of concomitant occurrence of AVMs and IAs that was successfully treated using the hybrid operation concept.

**Patient concerns:**

A 62-year-old man presented with sudden onset of severe headache, dizziness, nausea, and vomiting for 4 hours.

**Diagnosis:**

Computed tomography revealed SAH and a hematoma in the right frontal lobe. A computed tomographic angiogram also revealed a right frontal AVM with 3 IAs.

**Interventions:**

We used a hybrid operating room to successfully treat both AVMs and IAs.

**Outcomes:**

Two years of follow-up showed that the patients were well and performed their daily duties.

**Lessons:**

The hybrid operating room is an innovative, safe, and effective method for the treatment of AVMs with associated IAs, particularly high-grade AVMs and IAs with hemorrhage or SAH. Patients with concomitant AVMs and IAs have the highest chance of hemorrhage compared with those with AVM or IAs alone.

## 1 Introduction

The concept of hybrid surgery for the treatment of brain arteriovenous malformations (AVMs) with associated intracranial aneurysms (IAs) is still not widely practiced.^[1–5]^ The hybrid operating room allows for a combination of endovascular and surgical management of cerebrovascular lesions.^[1–5]^ This operating room uses interventional tools, such as temporary/permanent occlusion during clipping in a single procedure.^[2]^ This combined treatment option is currently the most appropriate for concomitant AVMs and IAs.^[1–5]^

IAs are detected in 7.5% to 23.5% of patients with AVMs.^[6–8]^ Patients with concomitant AVM and IA often have a higher chance of hemorrhage as a result of either AVM or IA rupture, although the precise risk is unclear.^[9]^ AVMs with associated IAs often originate from the feeding artery or within a proper AVM.^[6,7]^ The concomitant occurrence of these lesions is most likely based on a combination of congenital factors as well as augmented flow dynamics of the AVM.^[6,8,10]^ IAs that concomitantly occur with AVMs are categorized into arterial aneurysms (prenidal) or venous aneurysms (intranidal and postnidal) based on their relationships with the AVM nidus.^[11]^

The natural history of these concomitant lesions and appropriate treatment approaches are not yet well defined because the pathophysiology of the distinctive IA categories is poorly understood.^[2,7]^ Clinically, most patients with concomitant lesions are asymptomatic. Symptomatic patients often present with intracranial hemorrhage.^[11]^ These lesions are best detected with computed tomography angiography (CTA), magnetic resonance angiography (MRA), or digital subtraction angiography (DSA).^[12]^ Therefore, we present a rare case of concomitant occurrence of IAs and AVMs that we successfully treated using the hybrid operation concept.

## 2 Case report

A 62-year-old man presented with sudden onset of severe headache, dizziness, nausea, and vomiting for 4 hours prior to his admission to our neurosurgery department. He had no history of hypertension or diabetes mellitus. A general physical examination on admission did not yield much results. The neurological examination results were unremarkable. The Glasgow Coma Scale score on admission was 15 points, while the Hunts and Hess scale score was grade 1. Routine laboratory investigations were performed in the normal range. Electrocardiogram and chest radiography did not show any abnormalities.

**Figure 1:**
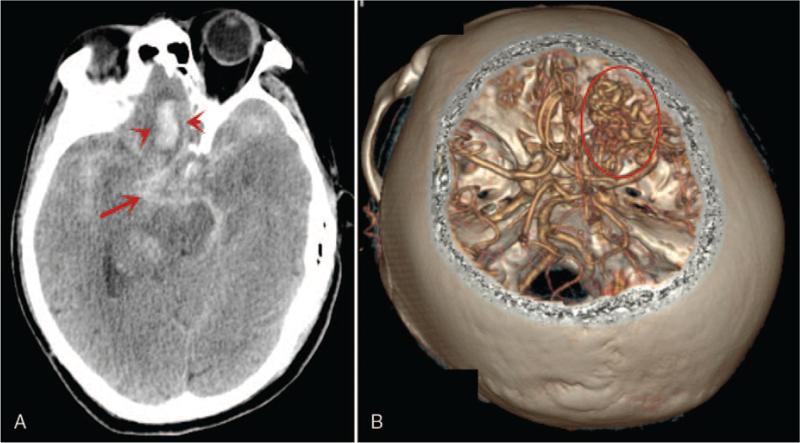
A and B: Are computed tomographic (CT) scan and computed tomographic angiogram (CTA). A: Subarachnoid hemorrhage (SAH) and hematoma in the right frontal lobe. B: Right frontal arteriovenous malformations (AVMs) with 3 intracranial aneurysms (IAs). Red circle = AVM; Red long arrow = SAH; red short arrows = hematoma in right frontal lobe.

**Figure 2:**
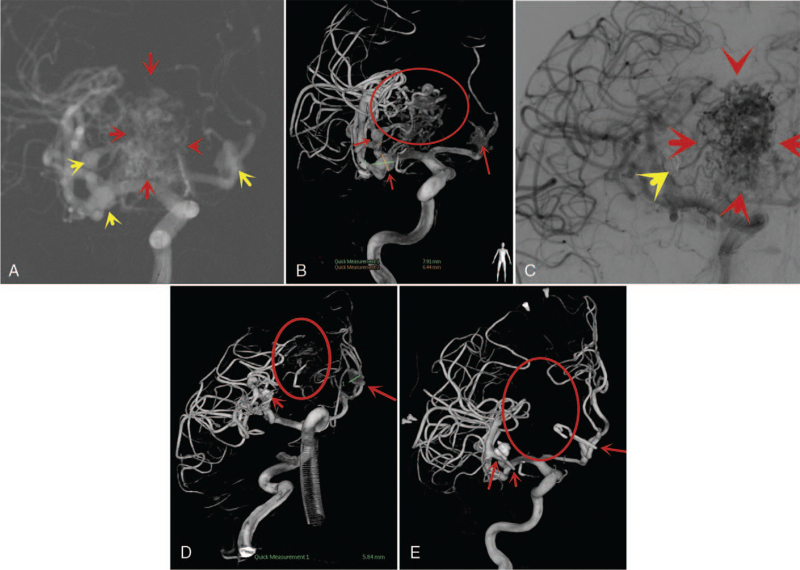
A–E: Are intraoperative image obtained during treatment at hybrid operating room. A: Digital subtraction angiography (DSA) showing the location of the AVM, 3 IAs, and feeders of the AVM. Red arrows, AVM; yellow arrows, intracranial aneurysms (IAs). B: Is intraoperative DSA showing a ruptured IA in A2 segment of the right anterior cerebral artery (ACA) and 2 other IAs in segments M1 and M2 of the right middle cerebral artery (MCA). Red circle, AVM; red arrows, intracranial aneurysms (IAs). C and D: Intraoperative DSA showing embolization of the 2 IAs with coils to essentially obliterate them, while arteries from which the AVM arose were embolized with onyx. C: Red arrows = AVM and yellow arrows = IA. D: Red circle = AVM; red arrows = IA. E: Intraoperative DSA showing total resection of the AVM and clip of the ACA aneurysm in situ. Red circle = location of AVM, red arrows = location of the IAs and clip.

Computed tomography (CT) revealed a subarachnoid hemorrhage (SAH) and hematoma in the right frontal lobe (Fig. 1A). CTA also revealed a right frontal AVM with 3 IAs (Fig. 1B). We opted to treat the patient in our hybrid operating room because of the complexity of dual pathologies. After obtaining informed consent for the operation, the patient was transferred to the hybrid operating room. After general anesthesia, we performed diagnostic DSA, which confirmed the location of the AVM, the 3 IAs, and the feeders of the AVM (Fig. 2A).

Intraoperatively, the ruptured IA was located in the A2 segment of the right anterior cerebral artery (ACA), whereas the other 2 were located in the M1 and M2 segments of the right middle cerebral artery (MCA) (Fig. 2B). The right anterior choroidal artery and aneurysmal arteries directly fed the AVM. In addition, venous drainage was performed via the cavernous sinus, sigmoid sinus, and transverse sinus. The 2 unruptured IAs were occluded with coils to essentially obliterate them, while the arteries from which the AVM arose were embolized with onyx (ev3, Irvine, CA) via the trans-arterial route (Fig. 2C and D). The ruptured ACA was tortuous and could not be embolized. After embolization of the IAs in the MCA, AVM was significantly reduced.

Subsequently, craniotomy was performed to resect the AVM and clip the ruptured IA in the ACA. Intraoperatively, we first identified and clipped the ruptured IA in the ACA. Next, we located the M1 and M2 segments of the right MCA as well as the right anterior choroidal artery, which were the dominant arterial feeders, and ligated them to minimize the blood supply to the AVM. In addition, we ligated the venous site of the AVM and subsequently resected the entire AVM along its borders, leaving behind the MCA. Intraoperative DSA revealed total resection of the AVM and an ACA IA clip in situ (Fig. 2E). After the operation, the patient regained consciousness without neurological deficits or seizures.

**Figure 3:**
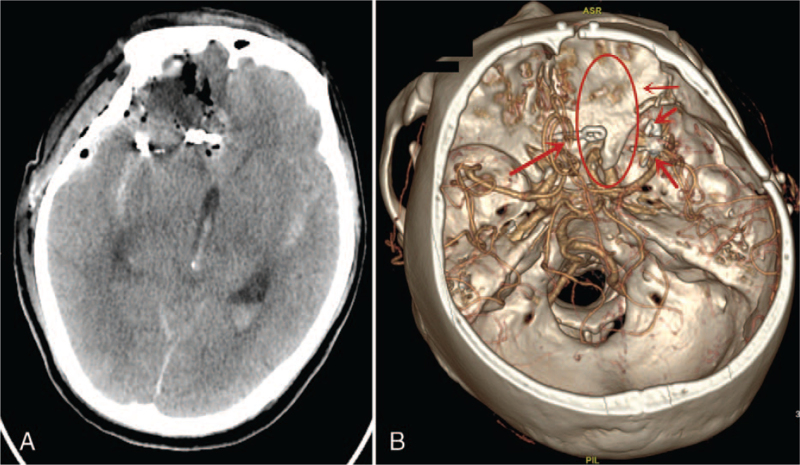
A and B: Are postoperative CT scan and CTA. A: Postoperative CT scan showing no intracranial hemorrhage. B: Postoperative CTA showing no AVM and a clip of the ACA in situ. Red circle and red thin arrow = location of AVM, red arrows = location of IAs and clip.

Postoperative CT revealed no intracranial hemorrhage (Fig. 3A). In addition, CTA showed no AVM or clip of the ACA IA in situ (Fig. 3B). The patient was discharged from the hospital 14 days after surgery. Scheduled follow-up visits to the outpatient department (OPD) showed no recurrence of symptoms. Two years of follow-up showed that the patients were well and performed their daily duties.

## 3 Discussion

The concomitant occurrence of AVMs and IAs has been reported previously.^[8,13,14]^ The concept of hybrid surgery for the treatment of AVM with associated IAs is uncommon.^[1–5]^ IAs are often characteristically detected within the AVM nidus or proximal feeding vessels.^[6]^ Elderly patients aged 55 to 60 years are more prone to SAH, which is twice as frequent in women than in men.^[15]^ Our patient was a 62-year-old man with concomitant AVM and IA. One of the IAs ruptured, resulting in acute SAH. AVMs, sporadic IAs, dural arteriovenous fistulas, and mycotic aneurysms are the most common potential sources of acute SAH.^[16–19]^ Acute SAH as a result of sporadic IA rupture is often due to minor continuous ruptures resulting in IA adherence to the adjacent arachnoid membrane or a high-pressure IA rupture that may cause pia-arachnoid perforation, leading to SAH.^[16,17]^

Brown et al^[20]^ observed 38% of SAH patients with presented with hemorrhagic onset in a study involving different bleeding patterns associated with the rupture of AVMs. Hartmann et al^[21]^ revealed that 30% of patients with ruptured AVMs presented with SAH, 23% presented with parenchymal hematoma, 16% presented with intraventricular hemorrhage (IVH), and 31% presented with combined hemorrhagic pattern. They further observed that in patients with SAH, 41% presented with focal deficits, 28% presented with IVH, and 3% of patients hesitated to have severe disability.^[21]^ Sturiale et al^[22]^ studied the relationship between nidus size and extension of the associated parenchymal hematoma and observed that nonparenchymal bleeding might worsen the prognosis of patients with hemorrhagic AVMs.

Studies have shown that patients with concomitant occurrence of AVMs and IAs often have approximately 7% to 10% chances of rupture compared with approximately 2% to 4% chances of in patients with AVMs without related aneurysms.^[6–8]^ Cagnazzo et al^[9]^ also observed in a meta-analysis that patients with concomitant AVMs and IAs were more likely to present with hemorrhage compared with the general AVM population (64% vs 50%). They indicated that, among patients with concomitant AVMs and IAs, the origin of the hemorrhage was the IA in 49% of patients and in 80% of patients, flow-related IAs were the most frequent source of aneurysmal hemorrhage.^[9]^ They further revealed that in patients with ruptured AVMs with associated IAs, the origin of the hemorrhage was the IA in 49.2% of patients, while in 45% of them, the AVM nidus was the source of the hemorrhage and the origin of bleeding was uncertain in 5.7% patients.^[9]^

The pathophysiology of AVM is depicted with a nidus composed of a conglomeration of venous tangles and loops.^[11,14]^ Venous drainage often originates at the nidus level with the arterial vessels ending just before, such that the arteriovenous shunt would arise slightly proximal to the nidus, as observed in surgical findings and pathologic studies.^[11,14]^ Furthermore, the function of the red veins is another key pathophysiological indicator.^[11,14]^ AVMs draining veins often contain arterialized blood and sustained arterial pressure.^[11,23]^ Their walls may be easily disrupted and mistaken for arteries.^[11,23]^ Moreover, both the arterial and venous AVM segments secrete the S2 marker, suggesting a contractile phenotype of smooth muscle cells. Thus, venous segments of brain AVMs can easily be mistaken for arterial components.^[11,24]^

There are 3 main theories regarding the causes of the concomitant occurrence of AVMs and IAs. Firstly, both lesions are congenital malformations.^[11,13]^ Second, their correlation may be spontaneous or coincidental.^[11,14]^ Third, IA may occur because of hemodynamic stress associated with augmented flow to the AVM.^[11,25]^ These theories do not contradict each other, and all 3 may be acceptable because distinctive types of concomitant occurrences of IAs and AVMs have been observed. It is worth noting that many classifications have been suggested for concomitant IA and AVM.^[8,11,26]^ These include prenidal, intranidal, and postnidal IAs.^[11]^ The prenidal types, which are entirely arterial lesions, are often subdivided into flow-unrelated, flow-related, and flow-related adjacent types according to their location in relation to the AVM nidus, whereas the latter types are completely intranidal and postnidal venous lesions.^[11]^ Prenidal IAs are often associated with the rate of blood flow and are usually found in AVM vessels.^[11]^ The 3 IAs in our patients were of the prenidal type (flow-related remote type) based on their location in relation to the AVM nidus.^[11]^

The key symptom in our patient was the sudden onset of severe headache, dizziness, nausea, and vomiting. When hemorrhage is suspected, CT is often performed to establish intracranial bleeding.^[12]^ If hemorrhage was established during CT evaluation, CTA was performed to determine the cause of the hemorrhage.^[12]^ CTA can show both AVMs and IAs. CTA is also capable of detecting the site of bleeding in prenidal, intranidal, and postnidal IA.^[12]^ In our patient, CT revealed SAH and hematoma in the right frontal lobe, while CTA revealed a right frontal AVM with 3 IAs. AVMs with associated IAs. MRI can detect shunts and evaluate the perinidal brain parenchyma.^[12]^ In addition, MRA is capable of detecting dilatation of arterial feeders depending on the shunt volume and flow-induced IAs along the feeding arteries.^[12]^ We did not perform MRI or MRA because we planned to manage the patient in our hybrid room, where we could perform diagnostic DSA.

DSA can be used to analyze AVM architecture, such as the nature and number of feeding arteries.^[12]^ DSA is also capable of showing the number of distinct compartments within the AVM, any intranidal or perinidal IA, the nature of the venous drainage, and related varices and stenoses.^[12]^ The DSA is a key component of a hybrid operating room. Thus, we opted to perform diagnostic DSA during the treatment of the patient via the endovascular route in our hybrid operating room. Our diagnostic DSA confirmed the location of the AVM, 3 IAs, and feeders of the AVM. Surgery and endovascular therapy are the main treatment options for AVMs and IA. Studies have shown that treatment with an AVM alone results in a regression of IAs on proximal feeding vessels.^[6,8,27]^

Coil embolization, Onyx or N-butyl cyanoacrylate (NBCA) embolization, and ethanol sclerotherapy are endovascular treatment modalities for AVM-related IA.^[6,28]^ Coil embolization is used to essentially obliterate the IA, whereas Onyx or NBCA embolization is frequently used for occlusion of the artery from which the IA arises.^[6]^ In some cases, Onyx and NBCA have been used to precisely obliterate IA with conservation of flow within the parent artery.^[6,28]^ Precipitation hydrophobic injectable liquid (PHIL) and SQUID are current liquid embolic agents that can be used to manage patients with AVMs with associated IAs.^[29]^

Several studies have demonstrated that proximal flow-related IAs, such as pedicle IAs, may drastically reduce or even vanish when they are not treated at the time of AVM obliteration.^[11,19,30]^ Miyasaka et al^[31]^ observed an overall regression rate of 14% for IAs with related AVMs after the AVMs were treated. Redekop et al^[8]^ also observed spontaneous regression of 50% of untreated IAs related to AVMs after gamma knife surgery for AVMs. They observed total resolution of flow related types and V-type IAs in 80% of patients when the AVMs were totally obliterated.

We utilized the hybrid operation concept to successfully treat both AVMs and IAs. Studies have shown that a combination of microsurgery, intraoperative DSA, and endovascular therapy in a hybrid operating room is an innovative, safe, and effective method for the treatment of AVMs, particularly high-grade AVMs.^[1–5,32]^ Nevertheless, there are no reports on the use of the hybrid operation concept in treating concomitant AVMs and multiple IAs. Identification of complications and augmented treatment goals are often achieved using intraoperative imaging during neurosurgery.^[2,32,33]^ Several studies have demonstrated that intraoperative DSA and near-infrared indocyanine green video angiography are capable of evaluating vascular flow during cerebrovascular surgery and contribute to safety and avoidance of severe complications during cerebrovascular procedures.^[2,33]^

The hybrid operating room was crucial in the management of our patient because we were able to assess the vasculature of the lesions intraoperatively. Intraoperatively, DSA clearly demonstrated the arterial supply of both lesions, as well as the draining sinus of the AVM. We were able to occlude the 2 unruptured IAs and embolize the feeding arteries of the AVM, after which we performed a craniotomy to resect the AVM and clipped the ruptured IA in a single operation. The hybrid operation concept minimized intraoperative blood loss and complications associated with the management of both AVMs and IAs. Intraoperative DSA was performed immediately after surgical resection of the AVM to confirm total AVM resection.

## 4 Conclusion

The hybrid operating room is an innovative, safe, and effective method for the treatment of AVMs and associated IAs, particularly high-grade AVMs and IAs with hemorrhage or SAH. Coil embolization was used to obliterate the IA, whereas Onyx embolization was used to occlude the artery from which the IA arose subsequent to craniotomy and resection of the AVM. Patients with concomitant AVMs and IAs had the highest chance of hemorrhage compared with those with AVM or IAs alone.

## Author contributions

All authors contributed to data collection, drafting, and critical revision of the paper and agree to be accountable for all aspects of the work. Seidu A. Richard wrote the manuscript.

**Conceptualization:** Fei Xie, Lin Huang, Yongqiang Ye, Jianqiang Hao, Janwei Lv, Seidu A. Richard.

**Data curation:** Fei Xie, Lin Huang, Yongqiang Ye, Jianqiang Hao, Janwei Lv, Seidu A. Richard.

**Formal analysis:** Fei Xie, Lin Huang, Yongqiang Ye, Jianqiang Hao, Seidu A. Richard.

**Funding acquisition:** Fei Xie.

**Investigation:** Janwei Lv.

**Methodology:** Fei Xie, Lin Huang, Yongqiang Ye, Jianqiang Hao, Seidu A. Richard.

**Resources:** Fei Xie, Lin Huang, Yongqiang Ye, Jianqiang Hao, Janwei Lv, Seidu A. Richard.

**Supervision:** Fei Xie.

**Writing – original draft:** Seidu A. Richard.

**Writing – review & editing:** Fei Xie, Lin Huang, Yongqiang Ye, Jianqiang Hao, Janwei Lv, Seidu A. Richard.
